# Veteran Treatments: PTSD Interventions

**DOI:** 10.3390/healthcare6030094

**Published:** 2018-08-06

**Authors:** Steven G. Koven

**Affiliations:** Urban Studies Institute, University of Louisville, 426 West Bloom Street, Louisville, KY 40208, USA; steven.koven@louisville.edu

**Keywords:** post-traumatic stress disorder, meta-analysis, veterans, treatment, interventions

## Abstract

Post-traumatic stress disorder (PTSD) has resulted in high social costs in terms of the lingering inability of veterans to adapt to societal norms. These costs accrue to individual veterans, their families, friends, and others. In addition, society suffers from the lost productivity of veterans. There is a need to pay greater attention to the extant literature regarding the effectiveness or ineffectiveness of various interventions. This study reviews the most relevant research regarding PTSD, veterans, interventions, treatment, counseling, job training and medication. Increasing awareness of the existing state of knowledge can lead to better targeting of resources and better health outcomes.

## 1. Introduction

Many veterans who are devoid of visible disabilities have acquired invisible scars that negatively affect them throughout their lifetimes. Both visible and invisible scars have severe repercussions for families, friends, communities and the collective society. Mental health disorders, divorce, alcoholism, drug abuse, homelessness, depression, unemployment, underemployment, and criminal activity represent some of the negative side effects of traumatic stress. Suicides and post-traumatic stress disorder (PTSD) are tangible indicators of the invisible wounds of military service [1,2,3].

Payments for PTSD have escalated in recent years. Some trace the escalation to a 2010 rule change by the U.S. Veterans Administration that is associated with an increase in the approval of PTSD claims. The 2010 rule dispenses with the need to corroborate that hostile military action produces stress disorder (Office of Public and Intergovernmental Affairs, 2010). Ambiguities and inconsistencies in diagnoses also contributed to rising costs. In a 2005 report, the Department of Veterans Affairs, Office of Inspector General concluded that in 25% of the PTSD cases reviewed, inconsistencies occurred in rating methods as well as the process of verifying evidence. Error rates ranged from a high of 40.7% (Maine) to a low of 11% (Oregon). Over the lifetimes of the veterans, the questionable payments approximated many billions of dollars [3].

PTSD claims increased rapidly between 1999 and 2004. According to a 2005 Veterans Affairs report, between 1999 and 2004 the number of total veterans receiving disability compensation grew by less than 15% while the number of PTSD cases in this time-period grew by almost 80%. Veteran compensation for PTSD expanded to represent more than 20% of all compensation payments [4].

The monetary payments that veterans receive are not the only costs that are associated with PTSD. Other costs include marital problems, family violence, raising children with behavioral difficulties, problems of trust, closeness, communication, drinking, intimacy, friendships, physical violence and drug abuse [3,5,6]. The social costs of PTSD are high. Given the high costs, it is useful to gain a greater understanding of the academic literature addressing PTSD. Such a review of existing literature provides a fuller understanding of what we know and do not know about PTSD.

This study describes the most relevant academic research according to Google Scholar criteria. Identified articles are indicative of currency and interest among scholars and the wider Internet audience. Some high-quality research may not be included in the identified articles because they do not comport with the Google Scholar criteria. Descriptions of veteran PTSD research included in this study, therefore, are not all inclusive of PTSD research. Identification prioritizes prevalence and circulation among other factors.

## 2. Methods

This study describes highly accessed PTSD research. This strategy is similar but not identical to meta-analysis in the sense that it pools various studies in an effort to make inferences. The medical definition of meta-analysis refers to a quantitative statistical analysis that is applied to separate but similar experiments of different and usually independent researchers and that involves pooling the data and using the pooled data to test the effectiveness of the results. More generally, researchers have applied the term meta-analysis to the analysis of prior analyses [7]. Researchers regard meta-analysis highly. Meta-analysis ranks at the top in the hierarchy of clinical evidence according to its freedom from various biases [8]. This study also employs content analysis. Content analysis refers to a method for studying documents and means of communication, which might be texts, pictures, audio or video. One of the key advantages of using content analysis is its non-invasive nature, in contrast to simulating social experiences or conducting surveys. Practices and philosophies of content analysis vary between academic disciplines. However, all content analysis studies involve systematic reading or observation of texts or artifacts that researchers assign labels to indicate the presence of meaningful pieces of content [9].

This paper combines aspects of content analysis and meta-analysis; it uses the google research tool for the selection of relevant studies. The criteria for the selection of the 20 articles (10 each for each of the two areas of investigation) includes a search of scholarly work using key words, a sorting based on relevance, and selection of the 10 most relevant scholarly articles for each avenue of inquiry. Google Scholar ranks relevance by multiple criteria. These criteria include weighing the full text of each document, where it was published, whom it was written by, as well as how often and how recently it has been cited in other scholarly literature. A previous study uses similar criteria in choosing research for meta-analysis [10]. This study ranks the most relevant manuscripts considering two foci of analysis. The first area of inquiry uses a search of the keywords PTSD, veterans, interventions and treatment. The second uses the keywords PTSD, veterans, counseling, job training and medication. The first search focuses on general veteran PTSD research. The second focuses more specifically on the type of treatments. Books are not included in the relevance rankings. Both groups of publications use PTSD and veterans in the content search. However, the distinction between the more general interventions used to identify the first group and the more specific terminology of counseling, job training and medication used to identify the second group was sufficiently distinctive to produce two entirely different cohorts. None of the identified articles appears in both groups.

Previous studies delineate methodologies for identifying prior research [11,12]. This study adds to the body of research that leverages electronic media for identifying relevant research. The study utilizes key terms PTSD, veterans, interventions, and treatment for identifying research based on criteria developed by Google Scholar, a highly visible source of data in the electronic age. In contrast, the study utilizes the terms counseling, job training and medication in an effort to target the specific type of intervention for veterans with PTSD. The goal of the search is to make inferences about the prevalence of obvious types of treatment.

## 3. Results

Table 1 delineates the 10 most relevant manuscripts for the keywords PTSD, veterans, intervention and treatment. The table identifies relevance, year published, authors, titles and publisher of these scholarly works.

As a whole, the studies delineated in Table 1 identify a wide variety of authors and publication outlets. Steven Southwick assists in two of the manuscripts. Articles appear in an array of journals; two manuscripts appear in the journal *Clinical Psychology Review*. Psychology journals are the major outlets for publication. With regard to the question of general treatment and intervention, two foci exist: (1) a treatment focus and (2) a problem focus.

Three of the 10 general treatment and intervention studies deal with meta-analysis [16] or controlled experiments [15,17]. Murphy, et al. (2009) study the awareness of the need to change among patients [17]. The authors find that poor response to PTSD treatment may be due not to inadequate interventions or biologically driven symptoms but from ambivalence or lack of awareness about the need to change. They posit that traumatized combat veterans may not see coping styles (e.g., social isolation, mistrust of others) as psychiatric symptoms but as functional strategies. The authors conclude that there is a need to focus on readiness to change. Ulmer, et al. (2011) found that newly developed interventions can address nightmare problems but do not fully remove all aspects of PTSD-related sleep difficulties [15].

Three articles describe innovative treatments/therapy for PTSD. Vujanovic et al. (2013) focuses on the application of meditation to trauma-related mental health struggles [21]. They note that as the utilization of “mindfulness-based” interventions increases, more research is necessary to determine how the intervention might alleviate psychological problems. Cukor et al. (2009) reviews emerging psychotherapeutic and pharmacologic interventions (including propranolol, ketamin, prazosin, and methylendioxymethaphetamine) for the treatment of PTSD [19]. Their paper states that the high rate of treatment failures for PTSD calls for the innovation and dissemination of alternative treatments. The authors review emerging interventions for the treatment of PTSD. They examine the evidence for a range of interventions, from social and family-based treatments to technological-based treatments and describe recent findings regarding novel pharmacologic approaches. The article gives special emphasis to virtual reality as a treatment.

Southwick et al. (2006) present “logotherapy” (healing through meaning) as an innovative adjunctive treatment for PTSD [22]. Logotherapy is future-oriented, focuses on personal strengths and places responsibility for change on the patient. The main tenets of logotherapy include “tragic optimism” or optimism in the face of human suffering guilt and certain death. Tragic optimism encompasses the human potential to transform suffering into human achievement and guilt into meaningful action. The authors view logotherapy as an adjunctive therapy, enhancing rather than supplanting other treatment approaches. They demonstrate how providers can apply logotherapy to the treatment of veterans with PTSD in a variety of therapeutic settings. The study concludes that through a variety of means, including Socratic dialogue, topical discussion using quotations, volunteerism, collective service projects, and group process, veterans can rediscover meaning in their lives. Veterans can see post-traumatic stress as a “heavy gift” and see themselves stronger because of their symptoms.

The final four general articles delineated in Table 1 link PTSD with specific problems that emanate from stress disorder. Bremner et al. (1996) address the effects of alcohol and substance abuse on PTSD patients [13]. They note that an increase in alcohol and substance abuse typically parallels the increase in symptoms of PTSD. Family and relationship problems are the focus of attention in two articles [14,18]. These studies describe numbing, anger, divorce, and severe relationship problems as aspects of PTSD. The final article explores cause of death among male veterans who receive treatment for PTSD. Authors find that behavioral causes (e.g., accidents, substance abuse, suicide, homicide or shooting by police) reduce the life expectancy of PTSD patients [19].

Table 2 describes the most relevant research inserting the key words PTSD, veterans, counseling, job training and medication. A variety of researchers publish in this area; however, the scholarship of Edna Foa is most prominent. Scholarship appears in multiple journals; the publication outlet *Journal of Traumatic Stress* is most prominent for the articles identified in Table 2.

Articles describe numerous treatment options. Hyer et al. (1996) discuss the effect of placing people in a novel setting, such as a wilderness [24]. The authors test whether an “Outward Bound Experience” of 5 days in a novel setting has positive effects on PTSD patients. They conclude that the “Outward Bound Experience” did not outperform inpatient programs although many veterans rated the experience positively. Another study (relevance 4) examine Eye Movement Desensitization and Reprocessing (EMDR) Therapy concluding that EMDR can produce significant improvements in reducing anxiety, anger, depression, isolation, intrusive thoughts, flashbacks, nightmares and relationship problems [26]. EMDR asks patients to recall distressing images while conducting actions such as side-to-side eye movements or hand tapping. The paper suggests that EMDR training is a more effective treatment for PTSD than those traditionally provided in an in-patient PTSD-specific program. A third study (relevance 9) explores the effectiveness of mindfulness-based stress reduction (MBSR) programs. MBSR uses a combination of meditation, body awareness, and yoga [31]. The authors conclude that MBSR can achieve significant improvements in PTSD symptoms.

In addition to these studies of various treatments, one manuscript (relevance 7) addresses prolonged exposure and three other types of treatments in rape victims [29]. The authors conclude that prolonged exposure (re-experiencing the traumatic event through remembering it and engaging with, rather than avoiding it) produces better outcomes on reducing PTSD symptoms than other techniques. Only one article (relevance 6) addresses the needs of veterans for vocational rehabilitation, independent living, and family support [28]. Authors provide a comprehensive listing of resources for veterans with traumatic brain injury and post-traumatic stress disorder.

The final five articles found in Table 2 (relevance 1,3,5,8,10) address Prolonged Exposure (PE) therapy in one manner or another [23,25,27,30,32]. Foa et al. (2005) specifically explored the impact of Prolonged Exposure therapy on female assault survivors [30]. The plethora of research on this treatment is testament to its currency and relevance. A consensus view is that Prolonged Exposure is effective in reducing the symptoms of PTSD in veterans who receive care in clinics. Studies indicate that prolonged exposure therapy has yielded gains infrequently or never seen in the past with PTSD patients [23], which is one of the success stories of clinical psychology and psychiatry [32] and has proven its effectiveness in the treatment of PTSD [25]. In addition, research finds that PE can effectively treat rape survivors [29,30] and is a well-established treatment for PTSD [27].

## 4. Discussion

A review of the general treatment and intervention studies (Table 1) reveals two basic streams of research. One describes treatments for PTSD; another describes the problems associated with PTSD. Relevant articles describe innovative approaches and techniques such as logotherapy, “mindfulness” and novel drugs. Recent research offers insights into how to mitigate the effects of PTSD. Keynan et al. (2016) argue that excluding veterans with combat PTSD (CPTSD) from eligibility for special recognition (such as the Purple Heart) strengthens their stigma and has detrimental implications for their wellbeing [33]. The authors contend that reclassifying PTSD to PTSI (posttraumatic stress injury) may mitigate the stigma of their wounds, increase their willingness to seek aid, and improve their chance to heal. Steenkamp et al. (2015) reviewed evidence from clinical trials of psychotherapies for PTSD in military and veteran populations. They found that two trauma therapies (cognitive processing therapy and prolonged exposure) are most frequently studied [34]. These treatments attained clinically meaningful symptom improvements; however, large proportions of patients retained their PTSD diagnosis after treatment.

A review of the more specific treatment and intervention literature (Table 2) focuses upon Prolonged Exposure (PE) as an effective treatment for PTSD. Some of the literature addresses relatively novel approaches such as mindfulness and Eye Movement Desensitization Reprogramming (EMDR). It is worth noting that despite keyword searches that utilize “counseling” and “job training”, only one article [28] has a focus on rehabilitation and offers practical recommendations for counseling professionals. A dearth of attention to the practical problems of veteran reintegration into society through employment or education seems to represent a deficiency in the literature. Poor employment prospects of PTSD patients may contribute to this relative lack of attention. The literature, however, is not completely devoid of employment-related studies. Ellison et al. (2018) found that veteran peer interventions can have positive effects with veterans subsequently spending greater amounts of time on education activities [35]. Amara et al. (2018) discovered that an array of factors such as severity of traumatic brain injury, drug abuse, age, education, and marital status were related to employment status of male and female post-9/11 veterans. Unemployment rates were similar for male and female veterans evaluated in the Veterans Health Administration for traumatic brain injury [36].

## 5. Conclusions

PTSD among veterans represents a significant problem for society, for families and individuals. Meta-analysis of relevant literature indicates that psychology and psychiatry academic journals are most prominent in pushing the boundaries of knowledge concerning PTSD treatments. The meta-analysis indicates some consensus on the viability of Prolonged Exposure therapy compared to other treatments. Given the high societal costs of PTSD, it is incumbent upon medical researchers to continue to explore treatment options. It is distressing, however, that the most relevant extant research does not assign greater attention to issues of job training, job placement, and social integration. Researchers should recognize that PTSD is a health issue but that job training, education, and job placement can also play an important role in veteran rehabilitation.

## Figures and Tables

**Table 1:** General treatment and intervention studies.

Relevance	Year	Author	Title	Journal
1	1996	Bremner	Chronic PTSD in Vietnam Combat Veterans [13]	American Journal of Psychiatry
		Southwick		
		Darnell		
		Charney		
2	2004	Galovsky	Psychological Sequelae of Combat Violence [14]	Aggression and Violent Behavior
		Lyons		
3	2011	Ulmer	A Multi-Component Cognitive-Behavioral Intervention for Sleep Disturbance in Veterans with PTSD [15]	Journal of Clinical Sleep Medicine
		Edinger		
		Calhoun		
4	2009	Bisson	Psychological treatments for chronic post-traumatic stress disorder [16]	British Journal of Psychiatry
		Mathews		
		Pilling		
5	2009	Murphy	Effect of a Motivation Enhancement Intervention [17]	Psychological Services
		Thompson		
		Murray		
		Rainey		
		Uddo		
6	2009	Monson	Military-related PTSD and intimate relationships [18]	Clinical Psychology Review
		Taft		
		Fredman		
7	2009	Cukor	Emerging Treatments for PTSD [19]	Clinical Psychology Review
		Spitalnik		
		Difede		
		Rizzo		
		Rothbaum		
8	2005	Drescher	Causes of Death among Male Veterans Who Receive Residential Treatment for PTSD [20]	Journal of Traumatic Stress
		Rosen		
		Burling		
		Foy		
9	2013	Vujanovic	Mindfulness in the Treatment of Posttraumatic Stress Disorder Among Military Veterans [21]	Spirituality in Clinical Practice
		Niles		
		Pietrefesa		
		Schmertz		
		Potter		
10	2006	Southwick	Logotherapy an Adjunctive Treatment for Chronic Combat-related PTSD [22]	American Journal of Psychotherapy
		Gilmartin		
		McDonough		
		Morrissey		

**Table 2:** Specific PTSD treatment and intervention studies.

Relevance	Year	Author	Title	Journal
1	2010	Karlin	Dissemination of evidence-based psychological treatments for posttraumatic stress disorder in the Veterans Health Administration [23]	Journal of Traumatic Stress
		Ruzek		
		Chard		
		Eftekhari		
		Monson		
		Hembree		
		Resick		
		Foa		
2	1996	Hyler	Effects of Outward Bound Experience as an adjunct to inpatient PTSD treatment of war veterans [24]	Journal of Clinical Psychology
		Boyd		
		Scurfield		
		Smith		
		Burke		
3	2009	Rauch	Prolonged exposure for PTSD in a Veterans Health Administration PTSD clinic [25]	Journal of Traumatic Stress
		Defever		
		Favorite		
		Duroe		
		Garrity		
		Matis		
		Leberzon		
4	1995	Silver	Treatment of Vietnam War Veterans with PTSD [26]	Journal of Traumatic Studies
		Brooks		
		Obenchain		
5	2002	Rothbaum	Exposure to therapy for posttraumatic stress disorder [27]	American Journal of Psychotherapy
		Schwartz		
6	2009	Burke	A New Disability for Rehabilitation Counselors [28]	Journal of Rehabilitation
		Degeneffe		
		Olney		
7	1991	Foa	Treatment of Posttraumatic Stress Disorder in Rape Victims [29]	Journal of Consulting and Clinical Psychology
		Rothbaum		
		Riggs		
		Murdock		
8	2005	Foa	Randomized Trial of Prolonged Exposure for Posttraumatic Stress Disorder with and without Cognitive Restructuring [30]	Journal of Consulting and Clinical Psychology
		Hembree		
		Cahill		
		Rauch		
		Riggs		
		Feeney		
		Elna		
9	2011	Kearney	Association of participation in a mindfulness program with measures of PTSD, depression and quality of life in a veteran sample [31]	Journal of Clinical Psychology
		McDermott		
		Malte		
		Martinez		
		Simpson		
10	2007	McNally	Mechanisms of exposure therapy: How neuroscience can improve psychological treatments for anxiety disorders [32]	Clinical Psychology Review
